# Founder effects on trans-generational dynamics of closed inbreeding lineages of the predatory mite *Phytoseiulus persimilis*

**DOI:** 10.1371/journal.pone.0215360

**Published:** 2019-04-11

**Authors:** Demet Çekin, Peter Schausberger

**Affiliations:** 1 Group of Arthropod Ecology and Behavior, Department of Crop Sciences, University of Natural Resources and Life Sciences, Vienna, Austria; 2 Department of Behavioural Biology, University of Vienna, Vienna, Austria; National Taiwan Normal University, TAIWAN

## Abstract

Both close inbreeding and distant outbreeding may reduce fitness below the level of individuals with intermediate parental relatedness. In the haplodiploid plant-inhabiting predatory mite *Phytoseiulus persimilis*, which is patchily distributed within and among host plants, fitness is indeed reduced in the short term, i.e. by a single generation of inbreeding. However, in the medium to long term (multiple generations), distant out-breeding should provide for favorable demographic founder effects in isolated populations. We tested this prediction in isolated experimental lineages founded by females mated to a sibling (close inbreeding), a male from the same population (intermediate relatedness) or a male from another population (distant outbreeding) and monitored lineage growth and persistence over four generations. Cross-generationally, lineages founded by distantly outbred females performed the best, i.e. produced the most descendants. However, this was solely due to superior performance from the F_2_ generation onwards, whereas in the F_1_ generation, lineages founded by females mated to males from their own population (intermediate relatedness) performed the best, as predicted from short-term in- and out-breeding depression effects. At the genetic level, this result was most likely due to distantly outbred founders introducing higher allelic variability and lower homozygosity levels, counterbalancing inbreeding depression, which inevitably occurs in isolated lineages, from the F_2_ generation onwards.

## Introduction

Founder effects, i.e. effects emerging from founding of populations/lineages by a subset of individuals of the parental population, have decisive influence on the occurrence and intensity of inbreeding depression [1,2]. Founder effects change the genetic composition, usually by reducing variation, of daughter populations/lineages relative to the parental population and are thus especially relevant for inbreeding by organisms structured in small and fragmented or locally isolated populations with limited genetic exchange [3,4].

The detrimental effects of close inbreeding were already studied and documented by Darwin (1876) [5] for various plant species. At the genetic level, increased homozygosity commonly results in fitness decrease, called inbreeding depression, with overdominance (general advantage of heterozygotes) or partial dominance (homozygosity of recessive deleterious alleles) as the underlying mechanisms [6–8]. At the species level, averaged across individuals and assuming partial dominance as causal mechanism, the deleterious effects of inbreeding depression are expected to be lower in haplodiploids than in diplodiploids. The reason is that in haplodiploid organisms with hemizygous males, recessive deleterious alleles should be more easily and more quickly purged than in diplodiploid organisms with heterozygous males [9–12]. Nonetheless, while at large inbreeding depression is indeed less severe in haplodiploid than in diplodiploid organisms [13–15], excessive inbreeding may cause significant negative fitness effects in haplodiploids as well [10,16–18]. Towards the distant end of the mate relatedness continuum, also excessive outbreeding may cause significant negative effects [19–21]. Common underlying reasons are behavioral incompatibilities at the phenotypic level, and genetic incompatibilities, break-down of co-adapted gene complexes and maladaptation at the genotypic level [7,22–23]. Therefore, according to optimal outbreeding theory [19], intermediate levels of mate relatedness should provide for the highest fitness [22,24].

Nonetheless, regarding the effects of mate relatedness on in- and out-breeding depression, the time scale and associated number of generations, i.e. distinction between short- (one generation) and medium- to long-term (multiple generations) effects, are decisive variables. Short-term uni-generational effects of mate relatedness on in- and out-breeding depression may differ from medium- to long-term trans-generational effects. The effects of chronic inbreeding, which inevitably occurs in isolated lineages founded by single or few individuals, because of increasing mate relatedness over time, should heavily depend on the genetic composition and diversity introduced by the founder individuals, i.e. their in- and outbreeding status [25–27]. Outbred founders should provide for increased genetic variability and/or decreased homozygosity, favoring trans-generational lineage growth and persistence. Under these circumstances, distant outbreeding by founders may be sub-optimal in the short term but may be more favorable than close and intermediate founder mate relatedness levels in the medium and long run [26,28].

We addressed this issue, that is, trans-generational effects of founder relatedness, in isolated experimental lineages of plant-inhabiting predatory mites *Phytoseiulus persimilis* Athias-Henriot (Acari: Phytoseiidae). *Phytoseiulus persimilis* are haplodiploid, with both females and males arising from fertilized eggs but males losing the paternal chromosome set during embryogenesis, a phenomenon dubbed pseudo-arrhenotoky [29]. *Phytoseiulus persimilis* are specialized predators of herbivorous spider mites, which are patchily distributed both at the local (within leaves and plants) and regional (among plants) spatial scale. Due to the spider mites’ patchy distribution pattern and mutual attraction, also their predators, *P*. *persimilis*, are patchily distributed within and among plants [30,31]. Local dispersal by *P*. *persimilis* occurs by ambulation whereas long-distance dispersal is passive using the wind [32]. *Phytoseiulus persimilis*’ prey specialization level, patchy distribution pattern and dispersal modes set the stage for the occurrence of in- and out-breeding and founder effects. However, knowledge of the consequences of in- and out-breeding by *P*. *persimilis* is very limited [33,34] and experimental studies addressing founder effects are lacking. *Phytoseiulus persimilis* has a short developmental time (5 to 6 d at 25°C) and high fecundity (up to 5 eggs/d at 25°C), resulting in intrinsic rates of increase (r_m_) >0.3/d [35,36]. Its specialized feeding habits and high population growth capacity render *P*. *persimilis* a highly efficacious, globally used biocontrol agent of spider mites [36]. Knowing the consequences of in- and out-breeding is imperative for optimizing *P*. *persimilis*’ mass-rearing and release strategies.

Recently, we scrutinized short-term (one generation) individual fitness effects of mate relatedness in *P*. *persimilis* [34]. In accordance with optimal outbreeding [19], we observed that an intermediate level of mate relatedness (same population) resulted in higher female fitness (higher fecundity) than excessively close (siblings) or distant (from geographically widely separated populations) mate relatedness [34]. The present study aimed at elucidating demographic founder effects, at graded levels of genetic relatedness between the founding female and her mate (sibling, same population and different population), in experimental lineages derived from two geographically widely separated populations of *P*. *persimilis*, Sicily and Greece. We hypothesized that chronically inbreeding lineages founded by outbred females, i.e. females mated to males from another population, produce more descendants and persist at higher abundance levels than lineages founded by sib-mated females and lineages founded by females mated to males from their own population. To experimentally increase the likelihood of occurrence and detectability of founder effects on inbreeding depression of *P*. *persimilis*, all lineages were subjected to food stress, i.e. held under limited prey conditions over 40 days, allowing about four generations (generation time [T] is ~10 to 11 d at 25°C) [36,37]. Under stressful conditions, in the short to medium term, recessive alleles are commonly more detrimental, and increase inbreeding depression effects, compared to benign conditions [26,38,39]. In the long term, chronically prevailing stress conditions may promote purging of deleterious recessive alleles and thereby lead to reduced inbreeding depression, as for example shown for fruit flies, *Drosophila melanogaster* [40,41].

## Materials and methods

### Experimental animals, population origins and rearing

Experimental animals derived from iso-female lines established from two laboratory-reared populations of *P*. *persimilis*. One population was founded by specimens collected in Sicily and the other by specimens collected in Greece [42]. *Phytoseiulus persimilis* is native at both sampling locations [43]; wide geographic separation suggests minimal genetic exchange, if occurring at all, between *P*. *persimilis* from Sicily and Greece. Each population was founded by 50 to 100 individuals and population sizes fluctuated between ~50 and a few hundred individuals. Both populations were maintained in the laboratory for about 6 to 7 years before starting the experiment.

In the laboratory, the predators were reared on separate artificial arenas consisting of plastic tiles (15 x 15 x 0.2 cm), delimited by water-saturated tissue paper, and resting on water-saturated foam cubes in plastic boxes (20 x 20 x 6 cm) half-filled with water. The predators were fed by adding leaves of common bean *Phaseolus vulgaris* L., infested by *Tetranychus urticae* Koch, onto the arena at two to three-day intervals [44,45]. *Tetranychus urticae* was reared on whole common bean plants *P*. *vulgaris*. For the experiments, the spider mites were brushed from infested leaves, using a paint brush or a mite brushing machine (BioQuip, CA, USA), onto glass plates, and then either singly picked up and placed into acrylic cages using a fine red marten’s hair brush or directly brushed onto detached leaf arenas or artificial tile arenas. Plants were grown at room temperature 23 ± 2°C and 16:8 h L:D photoperiod. All rearing and experimental units were kept in a climate chamber at 25 ± 1°C, 65 ± 5% RH and 16:8 h L:D photoperiod.

### Establishing iso-female lines

**Fig 1:**
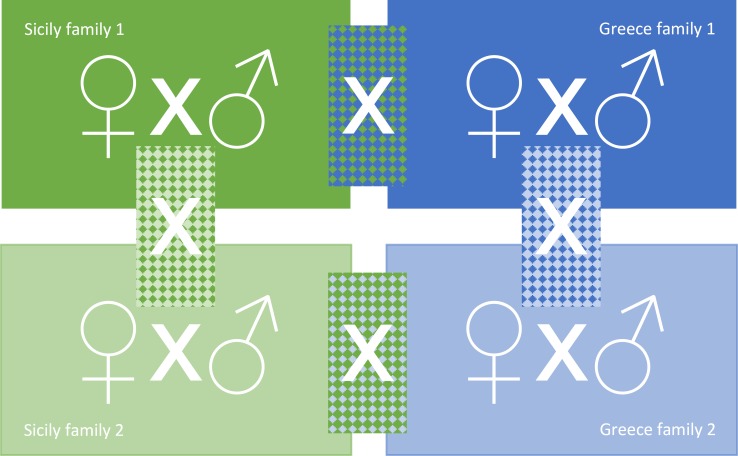
Crossing design used to establish three levels of in- and outbreeding in the founder females of the experimental lineages of *P*. *persimilis* (original figure [34]). Families were iso-female lines established from populations originating from Sicily and Greece. Mates came either from the same family (siblings), or from different families of the same population, or from different families from different populations.

**Fig 2:**
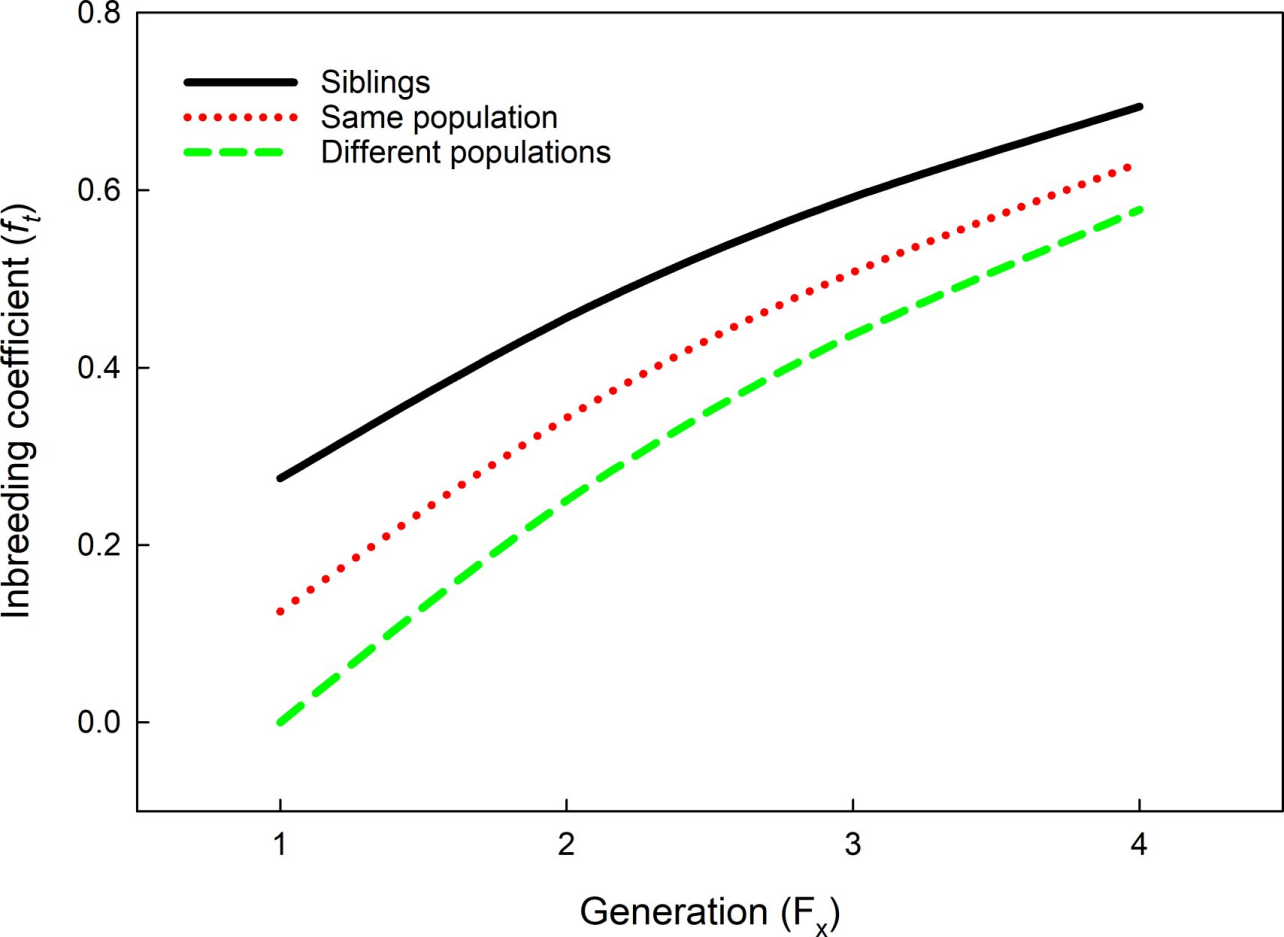
Predicted trans-generational development of Wright’s (1922) [48] inbreeding coefficient (*f*_*t*_) in chronically inbreeding experimental lineages of *P*. *persimilis* founded by females mated to a brother, a male from the same population or a male from another population (curve estimation using *Populus* 5.5 [51]). Assumed female to male inbreeding coefficients (*f*_*0*_) of the parental generation (F_0_) were 0.1 for sibling and same population mates and 0 for different population mates. Accordingly, *f*_*1*_ was 0, 0.125 and 0.275 for offspring (F_1_) of founders mated to males from different populations, the same population and siblings, respectively [50].

Founder females of the experimental lineages came from iso-female lines of *P*. *persimilis*, which were established as described by Atalay & Schausberger (2018) [34]. To establish iso-female lines, gravid females were randomly withdrawn from the stock populations and placed on spider mite-infested detached bean leaf arenas, each consisting of a trifoliate leaf placed upside down on a water-saturated foam cuboid, kept in a small plastic box (10 x 10 x 6 cm) half-filled with water, and delimited by water-saturated tissue paper. Eggs laid by the predators were transferred to new *T*. *urticae*-infested arenas and reared there until the deutonymphal stage. Deutonymphal females were singly isolated in cylindrical acrylic cages of 15 mm diameter and 3 mm height, laser-cut into rectangular acrylic plates (7.5 x 2.5 x 0.3 cm), closed with fine gauze at the bottom and a microscope slide on the upper side [46]. After reaching adulthood, each female was mated with a male randomly withdrawn from the stock population. Offspring of these couples represented a family (iso-female line) and were the individuals used in the experiment. Two families from each population (Sicily and Greece) were used in the experiment (Fig 1). Using the family design allowed comparing in- and out-breeding by individuals having the same set of alleles [39]. According to Nelson & Greef (2011) [47], the inbreeding coefficient (*f*_*t*_) [48] for offspring of full sib-mates (0.25) is almost equal in haplodiploids and diplodiploids [49]. In our experiment, we assumed inbreeding coefficients (*f*_*1*_) of 0 for offspring of distantly outbreeding couples (different populations), 0.125 for offspring of couples of the same population and 0.275 for offspring of sib couples. The latter two coefficients were based on the assumption of parental *f*_*0*_ = 0.1 because founders were withdrawn from closed and thus inbred laboratory populations [50]. The predicted increase in inbreeding coefficients of the three types of experimental lineages (Fig 2) was estimated with *Populus* 5.5 [51].

### Experimental procedure

The experiment was started by placing one virgin female and one male, at each level of genetic relatedness (siblings, same population, other population) from both populations, in acrylic cages, previously loaded with mixed spider mite stages, for 24 h (Fig 1). Cages were stored upside down on a grid in plastic boxes, the bottom of which was covered by water to warrant elevated humidity inside the cages [52]. After 24 h, the now mated females were singly placed on an acrylic arena, consisting of a plastic tile (15 x 15 x 0.2 cm) delimited by water-saturated tissue paper and resting on a water-saturated foam cuboid inside a plastic box (20 x 20 x 6 cm) half-filled with water. The predators were provided with predefined standardized amounts of mixed spider mite stages (i.e., brushed from three similarly sized bean leaves, each infested by ~50 to 80 *T*. *urticae*) in 3 d intervals. Over 40 days (allowing approximately four predator generations), we recorded the number and life stages of the predators (eggs, juveniles, adult females and adult males) in 5 d intervals. Each of the three levels of founder mate relatedness was replicated 13 to 22 times.

### Statistical analysis

Statistical analysis was carried out using IBM SPSS 23 (IBM; Armonk, NY, USA). For analysis, we used only counts of mobile predator life stages since accurately counting predator eggs was difficult on the arenas containing live prey and their webbing, and prey corpses. We used separate generalized estimating equations (GEE [53]; normal distribution, identity link function; M-dependent correlation matrix between observation days) to compare the influence of founder inbreeding level and population origin on lineage dynamics (abundance of mobile individuals) over the whole experimental period (40 d) and split into the first generation (<15 d) and from the onset of the second generation onwards (>10 d). Model selection was based on the QIC (quasi likelihood under independence model criterion) value. Mean generation time of *P*. *persimilis* is ~10 to 11 d at 25°C [36,37,54]. In case of a significant interaction term between founder inbreeding level and population origin, we conducted additional GEEs for the influence of mate relatedness on lineage dynamics within each population origin (Sicily and Greece). For post-hoc pairwise comparisons between founder inbreeding levels, we used least significant difference tests (LSDs).

## Results

**Fig 3:**
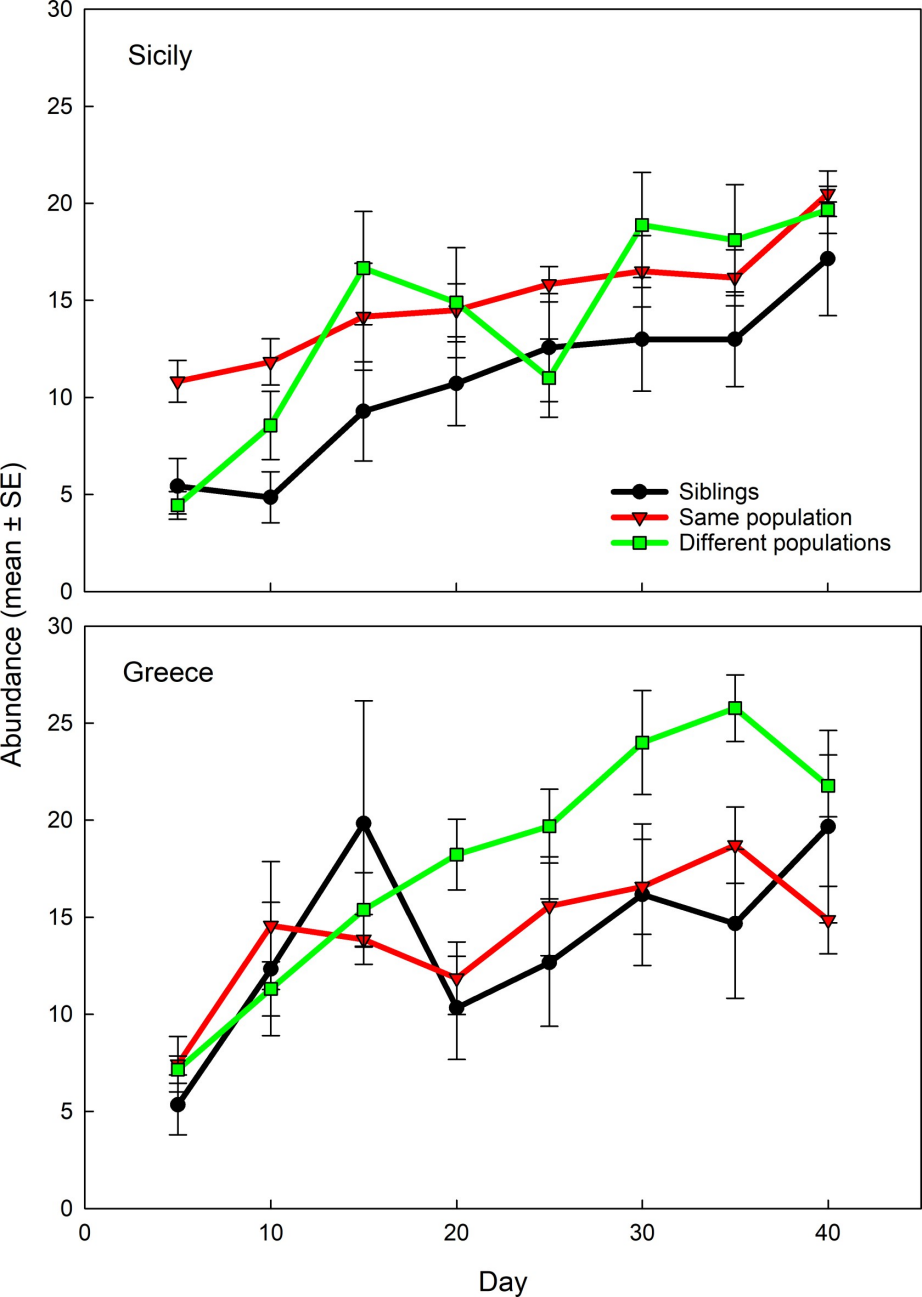
Trans-generational dynamics of experimental lineages founded by single *P*. *persimilis* females from Sicily and Greece. Founding females were mated to males with graded levels of genetic relatedness (siblings, same population, different populations) and lineage abundance (number of mobile mites) recorded over 40 days, corresponding to approximately four generations.

Mean abundance of predator lineages over the whole experimental period was significantly influenced by the interaction between founder inbreeding level and population origin (GEE: *Wald χ*_*2*_*^2^* = 6.402, *P* = 0.04), whereas neither founder inbreeding level (*Wald χ*_*2*_*^2^* = 2.749, *P* = 0.25) nor population origin (*Wald χ*_*1*_*^2^* = 0.281, *P* = 0.59) exerted significant main effects (Fig 3). Within either population origin, mean lineage abundance varied significantly with founder inbreeding level (GEE for Sicily: *Wald χ*_*2*_*^2^* = 7.683, *P* = 0.02; Greece: *Wald χ*_*2*_*^2^* = 6.319, *P* = 0.04). For the Sicily origin, lineages founded by sib-mated females reached significantly lower abundances (on average 20 to 30% lower) than lineages founded by females mated to males from the same population or to males from Greece (LSD: *P* < 0.05) (Fig 3). For the Greece origin, lineages founded by females mated to males from Sicily reached significantly higher abundances (on average 20% higher) than the population founded by females mated to males from the same population (LSD: *P* = 0.01) (Fig 3). All other pairwise comparisons were non-significant (*P* > 0.05). Two lineages founded by sib-mated females (one from Sicily and one from Greece) went extinct within the F_1_ generation (no individuals observed on day 15 and 20, respectively). All lineages founded by females mated to males from the same or the other population persisted until the end of the experiment.

**Fig 4:**
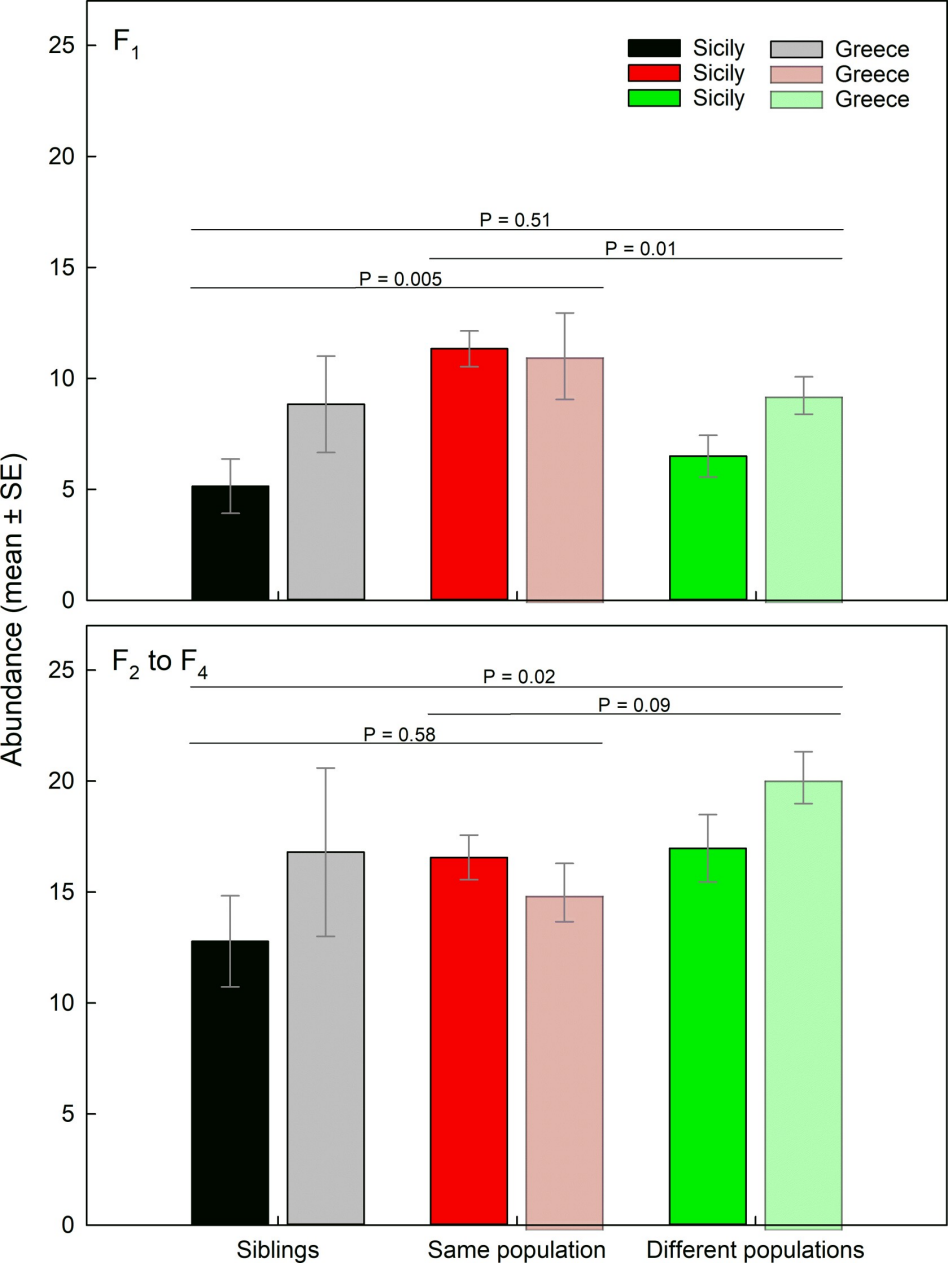
Mean generation-specific abundance of experimental lineages of *P*. *persimilis* founded by single females from Sicily or Greece, mated to males with graded levels of genetic relatedness (siblings, same population, different populations). Lineage abundance (number of mobile mites) was separately analyzed on days 5 and 10 of the experimental period (F_1_ generation; before F_1_ females started reproduction) and from day 15 onwards (F_2_ to F_4_ generations; after F_1_ females started reproduction). *P*-values refer to pairwise comparisons (LSD) between founder relatedness levels following GEE.

Mean lineage abundance of the F_1_ generation (until day 10) was significantly influenced by founder inbreeding level (GEE: *Wald χ*_*2*_*^2^* = 8.878, *P* = 0.01) and marginally by founder population origin (*Wald χ*_*1*_*^2^* = 3.028, *P* = 0.08) but not by the interaction of founder inbreeding level and population origin (*Wald χ*_*2*_*^2^* = 1.979, *P* = 0.37) (Fig 4). For both origins, Sicily and Greece, lineages founded by females mated to males from the same population reached significantly higher abundances (on average 30 to 35% higher) than lineages founded by sib-mated females (LSD: *P* = 0.007) and lineages founded by females mated to males from the other population (*P* = 0.01).

Mean lineage abundance from day 15 onwards (F_2_ to F_4_), i.e. after the onset of the F_2_ generation, was significantly influenced by founder inbreeding level (GEE: *Wald χ*_*2*_*^2^* = 6.602, *P* = 0.03) but not by founder population origin (*Wald χ*_*1*_*^2^* = 1.535, *P* = 0.21) and the interaction of founder inbreeding level and population origin (*Wald χ*_*2*_*^2^* = 4.121, *P* = 0.12) (Fig 4). For both origins, Sicily and Greece, lineages founded by females mated to males from the other population reached significantly higher abundances (on average 15 to 20% higher) than lineages founded by sib-mated females (LSD: *P* = 0.02) and lineages founded by females mated to males from the same population (*P* = 0.05).

## Discussion

Our study documents demographic founder effects in dependence of the founders’ in- and out-breeding status in the haplodiploid plant-inhabiting predatory mite *P*. *persimilis*. Trans-generationally, that is, over approximately four generations, closed lineages founded by distantly outbred females (mates coming from different populations) reached higher abundance levels than lineages founded by heavily (sibling mates) and lightly (mates coming from the same population) inbred females. However, time-dependent inter-lineage comparison revealed superior performance of lineages founded by outbred females only from the F_2_ generation onwards, whereas in the F_1_ generation lineages founded by females with intermediate mate relatedness levels (same population) performed better than lineages founded by sib-mated females and lineages founded by distantly outbred females (different populations).

Superior performance of lineages founded by females with intermediate mate relatedness in the F_1_ generation conforms to the predictions of optimal outbreeding theory [19] and was expected from beneficial short-term (F_1_ generation) individual fitness effects [34]. In the short term, females mated to males from the same population have a higher fecundity and longer oviposition period than females mated to siblings and males from another population [34]. Accordingly, in this study, lineages founded by females mated to males from the same population reached higher abundances in the F_1_ generation than lineages founded by sib-mated females and females mated to a male from the other population.

Throughout the experiment, all lineages were provided with the same limited amount of prey, hence food stress increased with increasing predator abundance in the course of the experiment. Limited food availability is a prime stressor during population development, fostering intraspecific competition and increasing individual starvation, retarding development, decreasing reproduction, and promoting propensities to dispersal and cannibalism. Higher frequencies of deleterious recessive alleles and homozygosity make inbred individuals commonly more sensitive to stressful conditions than outbred individuals [39]. Food stress was negligible in the F_1_ generation but increased from the F_2_ generation onwards. Beginning with the F_2_ generation, growth and abundance of sib-mated lineages and within population lineages was similar yet lower than growth and abundance of between population lineages. Obviously, within population lineages exceeded a critical inbreeding coefficient in the F_2_ generation (Fig 2), beyond which inbreeding depression effects became similar to those of lineages founded by sib-mated females. At the individual phenotypic level, genetic deficiencies associated with increasing inbreeding levels, may have adversely affected diverse inter-related physiological, life history and/or behavioral traits, such as decreasing general stress resilience, decreasing tolerance of energetic constraints, increasing mortality, prolonging development, decreasing fecundity, decreasing competitive abilities, and promoting the occurrence of cannibalism and dispersal tendencies [27,55,56,57]. Pinpointing the phenotypic mechanisms underlying the differences in performance of isolated lineages emerging from in- and out-bred founders requires additional experimental work.

Inter-lineage comparison from the F_2_ generation onwards revealed superior performance, that is, higher population growth and abundance, of lineages founded by outbred females, most likely because of higher allelic variability and lower homozygosity frequency alleviating inbreeding depression effects. Similarly, in the seminal experiments by Ayala (1965) [25], isolated populations of fruit flies *Drosophila serrata* emerging from genetically diverse founders reached higher growth rates and abundances than populations founded by genetically similar individuals. Saccheri et al. (1998) [58] identified increasing extinction risk with decreasing heterozygosity in natural populations of the Glanville fritillary butterfly *Melitaea cinxia*. Hildner, Soule, Min, & Foran (2003) [59] found an association between higher genetic variability and higher population growth rates in pocket gophers, *Thomomys bottae*. Agashe (2009) [60] and Agashe, Falk, & Bolnick (2011) [61] observed that populations of the red flour beetle *Tribolium castaneum*, founded by genetically diverse individuals, performed better (decreased extinction risk, faster adaptation in novel environment) than populations founded by genetically similar individuals. Apart from long-term outbred founder benefits, because of higher allelic diversity, outbreeding may also exert immediate positive fitness effects on population growth by heterosis, increasing hybrid vigor in the F_1_ generation [7,26,38]. For example, Drake (2006) [62] suggested heterosis being the prime genetic mechanism boosting population growth (dubbed “catapult effect”), and thus promoting establishment success in the wild, of red-necked pheasants *Phasianus colchicus*. Similarly, Wagner, Ochocki, Crawford, Compagnoni, & Miller (2017) [63] suggested heterosis causing a catapult effect in experimental lineages of the weevil *Callosobruchus maculatus*. However, in our study on *P*. *persimilis*, heterosis did not play a role because in the F_1_ generation lineages of outbred founders did worse than lineages of founders with intermediate level of relatedness.

Overall, our findings reveal that founder relatedness decisively influences the dynamics of chronically inbreeding lineages of the predatory mite *P*. *persimilis*. Our findings are highly relevant for the use of *P*. *persimilis* and other pseudo-arrhenotokous predatory mite species in augmentative and classical biological control of herbivorous mites, aiming at medium- to long-term predator establishment in novel environments. Our study predicts that colonization, establishment success and population growth and persistence of *P*. *persimilis* and other predatory mites should be higher when releasing outbred founders [64]. Answering for how long such founder effects will last and whether purging allows populations founded by inbred founders to catch up requires further investigations

## Supporting information

S1 Table Raw data of the experiment. (XLSX)
